# Supplementary figures and images for: A peptide-neurotensin conjugate that crosses the blood-brain barrier induces pharmacological hypothermia associated with anticonvulsant, neuroprotective, and anti-inflammatory properties following status epilepticus in mice
Source: eLife. 2025 Mar 28;13:RP100527. doi: 10.7554/eLife.100527 (PMC11952754; doi:10.7554/eLife.100527)

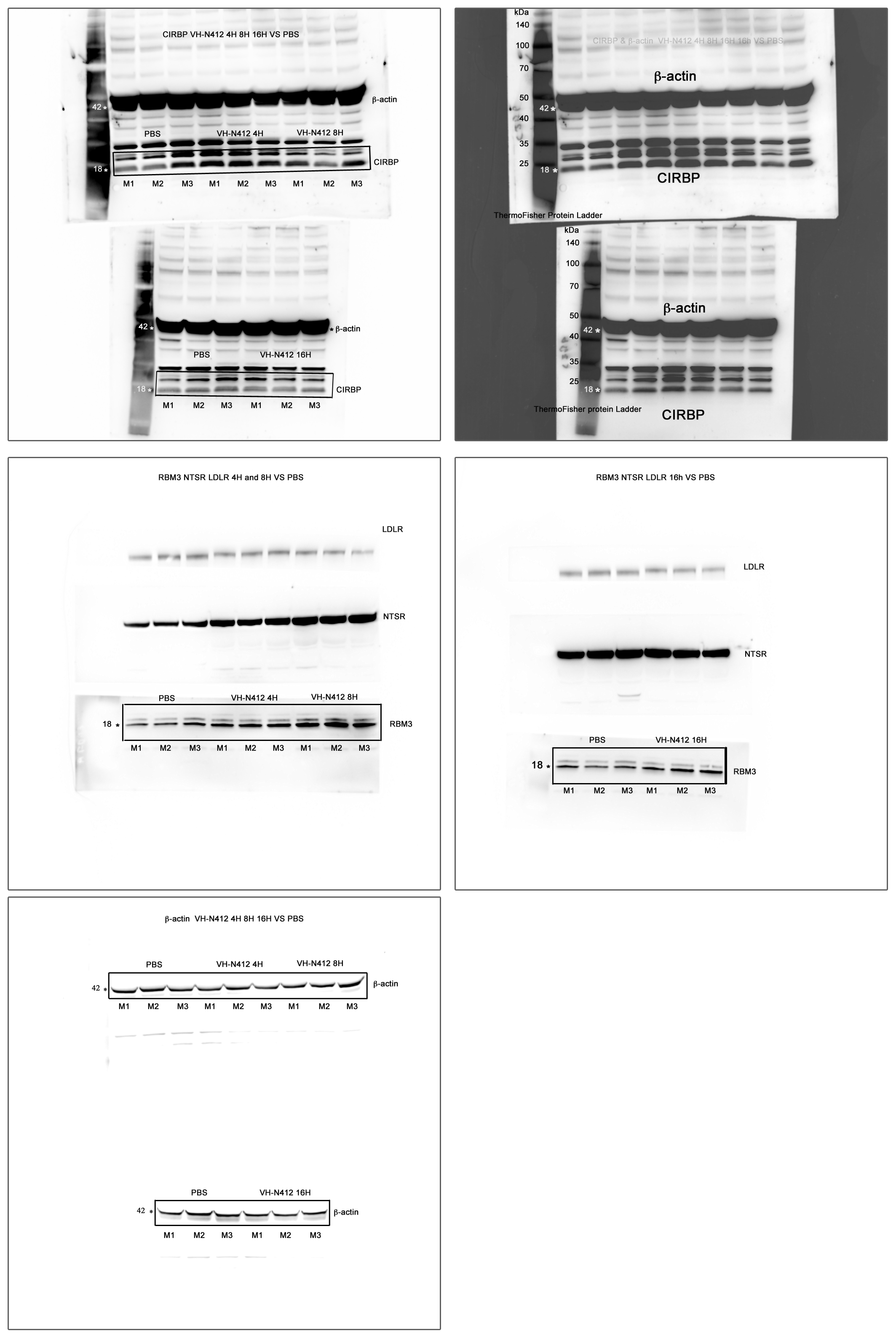

Supplement: Figure 9—source data 1. — Molecular weight ladders (black) depict separation range. Black boxes represent cropping as shown in Figure 9B. Membranes were physically cut (or not) following transfer and immunoblotted with different antibodies. White stars indicate the approximate size of different proteins of interest. Raw images are available on Dryad at https://doi.org/10.5061/dryad.nzs7h451x. [file elife-100527-fig9-data1.tif]
