# Supplementary material for: A peptide-neurotensin conjugate that crosses the blood-brain barrier induces pharmacological hypothermia associated with anticonvulsant, neuroprotective, and anti-inflammatory properties following status epilepticus in mice
Source: eLife. 2025 Mar 28;13:RP100527. doi: 10.7554/eLife.100527 (PMC11952754; doi:10.7554/eLife.100527)
Supplement: Supplementary file 3. [file elife-100527-supp3.docx]

Supplementary File 3. Comparison of mean body temperature changes between SHAM, SE, SE + VH-N412, SE + NT (8-13) and SE + DZP

| Mean body temperature changes (°C) ± SEM *P* Tukey’s test |
| --- |

-30 min

SHAM vs SE 38,64±0,20 vs 38,73±0,20 ns

SHAM vs SE+VH-N412 38,64±0,20 vs 38,58±0,22 ns

SHAM vs SE+NT(8-13) 38,64±0,20 vs 39,11±0,29 ns

SHAM vs SE+DZP 38,64±0,20 vs 39,24±0,28 ns

SE vs SE+VH-N412 38,73±0,21 vs 38,58±0,22 ns

SE vs SE+NT(8-13) 38,73±0,21 vs 39,11±0,29 ns

SE vs SE+DZP 38,73±0,21 vs 39,24±0,28 ns

SE+VH-N412 vs SE+NT(8-13) 38,58±0,22 vs 39,11±0,29 ns

SE+VH-N412 vs SE+DZP 38,73±0,21 vs 39,24±0,28 ns

0 min

SHAM vs SE 38,64±0,25 vs 39,08±0,40 ns

SHAM vs SE+VH-N412 38,64±0,25 vs 39,23±0,45 ns

SHAM vs SE+NT(8-13) 38,64±0,25 vs 40,00±0,28 ns

SHAM vs SE+DZP 38,64±0,25 vs 40,30±0,31 ns

SE vs SE+VH-N412 39,08±0,40 vs 39,23±0,45 ns

SE vs SE+NT(8-13) 39,08±0,40 vs 40,00±0,28 ns

SE vs SE+DZP 39,08±0,40 vs 40,30±0,31 ns

SE+VH-N412 vs SE+NT(8-13) 39,23±0,45 vs 40,00±0,28 ns

SE+VH-N412 vs SE+DZP 39,23±0,45 vs 40,30±0,31 ns

30 min

SHAM vs SE 38,38±0,15 vs 38,62+0,37 ns

SHAM vs SE+VH-N412 38,38±0,15 vs 36,50±0,34 ns

SHAM vs SE+NT(8-13) 38,38±0,15 vs 38,58±0,39 ns

SHAM vs SE+DZP 38,38±0,15 vs 36,10±0,44 ns

SE vs SE+VH-N412 38,62±0,37 vs 36,50±0,34 <0,01

SE vs SE+NT(8-13) 38,62±0,37 vs 38,58±0,39 ns

SE vs SE+DZP 38,62±0,37 vs 36,10±0,44 <0,01

SE+VH-N412 vs SE+NT(8-13) 38,62±0,37 vs 38,58±0,39 <0,01

SE+VH-N412 vs SE+DZP 38,62+0,37 vs 36,10+0,44 ns

60 min

SHAM vs SE 38,12±0,30 vs 39,43±0,30 ns

SHAM vs SE+VH-N412 38,12±0,30 vs 35,27±0,32 <0,01

SHAM vs SE+NT(8-13) 38,12+0,30 vs 38,30+0,54 ns

SHAM vs SE+DZP 38,12+0,30 vs 34,80+0,41 <0,01

SE vs SE+VH-N412 39,43±0,30 vs 35,27±0,32 <0,01

SE vs SE+NT(8-13) 39,43±0,30 vs 38,30±0,54 ns

SE vs SE+DZP 39,43±0,30 vs 34,80±0,41 <0,01

SE+VH-N412 vs SE+NT(8-13) 35,27±0,32 vs 38,30±0,54 <0,01

SE+VH-N412 vs SE+DZP 35,27±0,32 vs 34,80±0,41 ns

90 min

SHAM vs SE 37,88±0,41 vs 39,08±0,23 ns

SHAM vs SE+VH-N412 37,88±0,41 vs 35,07±0,41 <0,01

SHAM vs SE+NT(8-13) 37,88±0,41 vs 37,78±0,49 ns

SHAM vs SE+DZP 37,88±0,41 vs 34,38±0,60 <0,01

SE vs SE+VH-N412 39,00±0,23 vs 35,07±0,41 <0,01

SE vs SE+NT(8-13) 39,08±0,23 vs 37,78±0,49 ns

SE vs SE+DZP 39,08±0,23 vs 34,38±0,60 <0,01

SE+VH-N412 vs SE+NT(8-13) 35,07±0,41 vs 37,78±0,49 <0,01

SE+VH-N412 vs SE+DZP 35,07+0,41 vs 34,38±0,60 ns

120 min

SHAM vs SE 37,78±0,44 vs 37,78±0,44 ns

SHAM vs SE+VH-N412 37,78±0,44 vs 34,74±0,43 <0,01

SHAM vs SE+NT(8-13) 37,78±0,44 vs 37,53±0,39 ns

SHAM vs SE+DZP 37,78±0,44 vs 34,32±0,86 <0,01

SE vs SE+VH-N412 37,78±0,44 vs 34,74±0,43 <0,01

SE vs SE+NT (8-13) 37,78±0,44 vs 37,53±0,39 ns

SE vs SE+DZP 37,78±0,44 vs 34,32±0,86 <0,01

SE+VH-N412 vs SE+NT(8-13) 34,74±0,43 vs 37,53±0,39 <0,01

SE+VH-N412 vs SE+DZP 34,74+0,43 vs 34,32±0,86 ns

150 min

SHAM vs SE 37,92±0,30 vs 38,44±0,22 ns

SHAM vs SE+VH-N412 37,92±0,30 vs 35,10±0,42 <0,01

SHAM vs SE+NT(8-13) 37,92±0,30 vs 37,40±0,37 ns

SHAM vs SE+DZP 37,92±0,30 vs 34,72±0,89 <0,01

SE vs SE+VH-N412 38,44±0,22 vs 35,10±0,42 <0,01

SE vs SE+NT (8-13) 38,44±0,22 vs 37,40±0,37 ns

SE vs SE+DZP 38,44±0,22 vs 34,72±0,89 <0,01

SE+VH-N412 vs SE+NT(8-13) 35,10±0,42 vs 37,40±0,37 <0,01

SE+VH-N412 vs SE+DZP 35,10±0,42 vs 34,72±0,89 ns

SHAM (n=5 mice) ; SE (n=16 mice) ; VH-N412 (n=20) ; SE+NT(8-13)(n=14) ; SE+DZP (n=5 mice) ; ns, not significant.

Supplementary File 3: Ferhat et al.
