# Supplementary material for: A peptide-neurotensin conjugate that crosses the blood-brain barrier induces pharmacological hypothermia associated with anticonvulsant, neuroprotective, and anti-inflammatory properties following status epilepticus in mice
Source: eLife. 2025 Mar 28;13:RP100527. doi: 10.7554/eLife.100527 (PMC11952754; doi:10.7554/eLife.100527)
Supplement: Supplementary file 4. [file elife-100527-supp4.docx]

Supplementary File 4. Comparison of mean seizure intensity score between SHAM, SE, SE + VH-N412, SE + NT (8-13) and SE + DZP

| Mean seizure intensity score ± SEM *P* Tukey’s test |
| --- |

-30 min

SHAM vs SE 0,00±0,00 vs 5,19±0,10 <0,01

SHAM vs SE+VH-N412 0,00±0,00 vs 5,30±0,11 <0,01

SHAM vs SE+NT(8-13) 0,00±0,00 vs 5,00±0,00 <0,01

SHAM vs SE+DZP 0,00±0,00 vs 5,00±0,00 <0,01

SE vs SE+VH-N412 5,19±0,10 vs 5,30±0,11 ns

SE vs SE+NT(8-13) 5,19±0,10 vs 5,00±0,00 ns

SE vs SE+DZP 5,19±0,10 vs 5,00±0,00 ns

SE+VH-N412 vs SE+NT(8-13) 5,30±0,11 vs 5,00±0,00 ns

SE+VH-N412 vs SE+DZP 5,30±0,11 vs 5,00±0,00 ns

0 min

SHAM vs SE 0,00±0,00 vs 5,25±0,17 <0,01

SHAM vs SE+VH-N412 0,00±0,00 vs 5,40±0,11 <0,01

SHAM vs SE+NT(8-13) 0,00±0,00 vs 5,14±0,10 <0,01

SHAM vs SE+DZP 0,00±0,00 vs 5,00±0,00 <0,01

SE vs SE+VH-N412 5,25±0,17 vs 5,40±0,11 ns

SE vs SE+NT(8-13) 5,25±0,17 vs 5,14±0,10 ns

SE vs SE+DZP 5,25±0,17 vs 5,00±0,00 ns

SE+VH-N412 vs SE+NT(8-13) 5,40±0,11 vs 5,14±0,10 ns

SE+VH-N412 vs SE+DZP 5,40±0,11 vs 5,00±0,00 ns

30 min

SHAM vs SE 0,00±0,00 vs 5,38±0,15 <0,01

SHAM vs SE+VH-N412 0,00±0,00 vs 1,97±0,36 ns

SHAM vs SE+NT(8-13) 0,00±0,00 vs 4,71±0,46 <0,01

SHAM vs SE+DZP 0,00±0,00 vs 0,80±0,80 ns

SE vs SE+VH-N412 5,38±0,15 vs 1,97±0,36 <0,01

SE vs SE+NT(8-13) 5,38±0,15 vs 4,71±0,46 ns

SE vs SE+DZP 5,38±0,15 vs 0,80±0,80 <0,01

SE+VH-N412 vs SE+NT(8-13) 1,97±0,36 vs 4,71±0,46 <0,01

SE+VH-N412 vs SE+DZP 1,97±0,36 vs 0,80±0,80 ns

60 min

SHAM vs SE 0,00±0,00 vs 5,37±0,15 <0,01

SHAM vs SE+VH-N412 0,00±0,00 vs 1,57±0,32 ns

SHAM vs SE+NT(8-13) 0,00±0,00 vs 4,85±0,25 <0,01

SHAM vs SE+DZP 0,00±0,00 vs 0,80±0,80 ns

SE vs SE+VH-N412 5,37±0,15 vs 1,57±0,32 <0,01

SE vs SE+NT(8-13) 5,37±0,15 vs 4,85±0,25 ns

SE vs SE+DZP 5,37±0,15 vs 0,80±0,80 <0,01

SE+VH-N412 vs SE+NT(8-13) 1,57±0,32 vs 4,85±0,25 <0,01

SE+VH-N412 vs SE+DZP 1,57±0,32 vs 0,80±0,80 ns

90 min

SHAM vs SE 0,00±0,00 vs 5,43±0,13 <0,01

SHAM vs SE+VH-N412 0,00±0,00 vs 1,36±0,34 ns

SHAM vs SE+NT(8-13) 0,00±0,00 vs 4,92±0,25 <0,01

SHAM vs SE+DZP 0,00±0,00 vs 0,40±0,40 ns

SE vs SE+VH-N412 5,43±0,13 vs 1,36±0,34 <0,01

SE vs SE+NT(8-13) 5,43±0,13 vs 4,92±0,25 ns

SE vs SE+DZP 5,43±0,13 vs 0,40±0,40 <0,01

SE+VH-N412 vs SE+NT(8-13) 1,36±0,34 vs 4,92±0,25 <0,01

SE+VH-N412 vs SE+DZP 1,36±0,34 vs 0,40±0,40 ns

120 min

SHAM vs SE 0,00±0,00 vs 5,18±0,14 <0,01

SHAM vs SE+VH-N412 0,00±0,00 vs 1,10±0,27 ns

SHAM vs SE+NT(8-13) 0,00±0,00 vs 4,85±0,23 <0,01

SHAM vs SE+DZP 0,00±0,00 vs 0,00±0,00 ns

SE vs SE+VH-N412 5,18±0,14 vs 1,10±0,27 <0,01

SE vs SE+NT(8-13) 5,18±0,14 vs 4,85±0,23 ns

SE vs SE+DZP 5,18±0,14 vs 0,00±0,00 <0,01

SE+VH-N412 vs SE+NT(8-13) 1,10±0,27 vs 4,85±0,23 <0,01

SE+VH-N412 vs SE+DZP 1,10±0,27 vs 0,00±0,00 ns

150 min

SHAM vs SE 0,00±0,00 vs 5,25±0,11 <0,01

SHAM vs SE+VH-N412 0,00±0,00 vs 1,26±0,27 ns

SHAM vs SE+NT(8-13) 0,00±0,00 vs 4,85±0,23 <0,01

SHAM vs SE+DZP 0,00±0,00 vs 0,00±0,00 ns

SE vs SE+VH-N412 5,25±0,11 vs 1,26±0,27 <0,01

SE vs SE+NT(8-13) 5,25±0,11 vs 4,85±0,23 ns

SE vs SE+DZP 5,25±0,11 vs 0,00±0,00 <0,01

SE+VH-N412 vs SE+NT(8-13) 1,26±0,27 vs 4,85±0,23 <0,01

SE+VH-N412 vs SE+DZP 1,26±0,27 vs 0,00±0,00 ns

SHAM (n=5 mice) ; SE (n=16 mice) ; VH-N412 (n=20) ; SE+NT(8-13)(n=14) ; SE+DZP (n=5 mice) ; ns, not significant.

Supplementary File 4: Ferhat et al.
